# Extraskeletal Osteosarcoma of the Orbit

**DOI:** 10.1080/13577149878082

**Published:** 1998-06

**Authors:** Rojymon Jacob, Elizabeth Abraham, Rema Jyothirmayi, Madhavan Krishnan Nair

**Affiliations:** 1 Department of Radiotherapy Regional Cancer Centre Trivandrum Kerala India; 2 Department of Pathology Regional Cancer Centre Trivandrum Kerala India; 3 Department of Radiotherapy Royal Marsden Hospital NHS Trust Fulham Road London SW3 6JJ UK

## Abstract

*Patient.* We report a 22-year-old male presenting with extraskeletal osteosarcoma of the orbit.

*Discussion.* Extra skeletal osteosarcomas are uncommon tumours, usually arising from the lower extremities or girdle. These are aggressive tumours with high metastatic potential and poor outcome. Optimal treatment is undefined, and the role of radical surgery, radiotherapy and aggressive chemotherapy is currently being evaluated. The orbit is a rare site for extraskeletal osteosarcoma, with the only previous case reported in an 11-year-old male, who was irradiated in infancy for a retinoblastoma.


*Sarcoma* (1998) **2**, 121–124

CASE REPORT

### Extraskeletal osteosarcoma of the orbit

ROJYMON JACOB,[1]* ELIZABETH ABRAHAM,[2] REMA JYOTHIRMAYI,[1]* & MADHAVAN KRISHNAN NAIR[1]

[1]*Department of Radiotherapy, &* [2]*Department of Pathology, Regional Cancer Centre, Trivandrum, Kerala, India*

**Abstract**
*Patient.* We report a 22-year-old male presenting with extraskeletal osteosarcoma of the orbit.
*Discussion.* Extra skeletal osteosarcomas are uncommon tumours, usually arising from the lower extremities or girdle. These are aggressive tumours with high metastatic potential and poor outcome. Optimal treatment is undefined, and the role of radical surgery, radiotherapy and aggressive chemotherapy is currently being evaluated. The orbit is a rare site for extraskeletal osteosarcoma, with the only previous case reported in an 11-year-old male, who was irradiated in infancy for a retinoblastoma.

**Key words**: *extraskeletal, osteosarcoma, orbit.*

### Case report

A twenty-two-year-old man presented with a 1-year history of gradually increasing swelling of the left eye and occasional pain. On the whole, patient was well nourished and had no evidence of lymphnode enlargement. There was proptosis of the left eye with oedema in the upper eyelid. The conjunctiva showed mild congestion, and the cornea, anterior chamber and lens were clinically normal. Vision and ocular movements were normal. There was no evidence of raised intra-cranial pressure, meningeal irritation or focal neurological deficits. All other systems were normal.

Blood count and serum chemistry were normal. CT scan of the head revealed a well-circumscribed homogeneous mass in the supero-medial aspect of the left orbit, with non-homogeneous contrast enhancement (Fig. 1). The mass appeared separate from the globe and the bony orbital wall. On MRI, the lesion was homogeneously hypo-intense on T1- and hyper-intense on T2-weighted images. The patient underwent left frontal transcranial orbitotomy and complete excision of the orbital tumour. At surgery the tumour was found not attached to the orbital walls or extra-ocular muscles. It was separated by blunt dissection and entirely removed with-

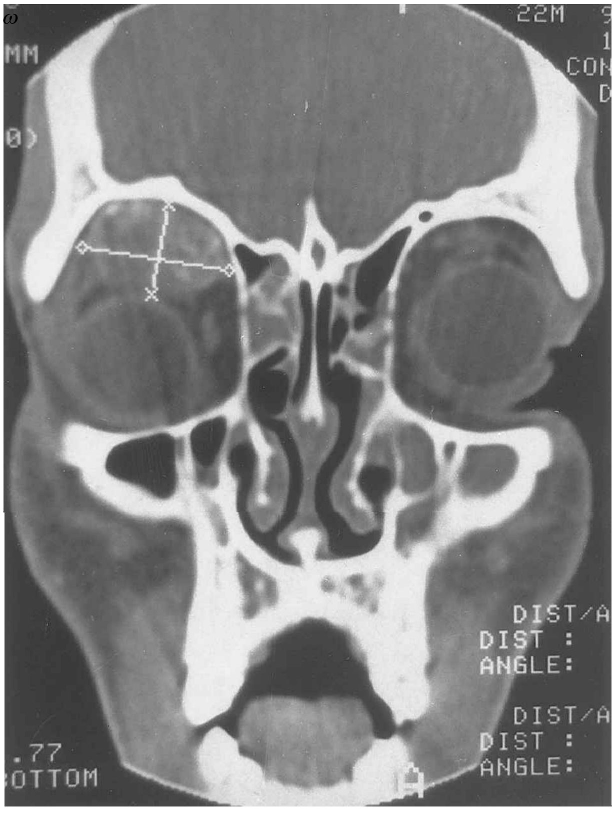

**Fig. 1.** *CT scan of the head showing the intra-orbital tumour.*

Correspondence to: R. Jacob, Department of Radiotherapy, Royal Marsden Hospital NHS Trust, Fulham Road, London, SW3 6JJ, UK. Fax: +44 171 3490786; E-mail: rojymon@icr.ac.uk.
*Present address: Department of Radiotherapy, Royal Marsden Hospital, Fulham Road, London SW3 6JJ, UK.

1357-714X/98/020121-04 ©1998 Carfax Publishing Ltd

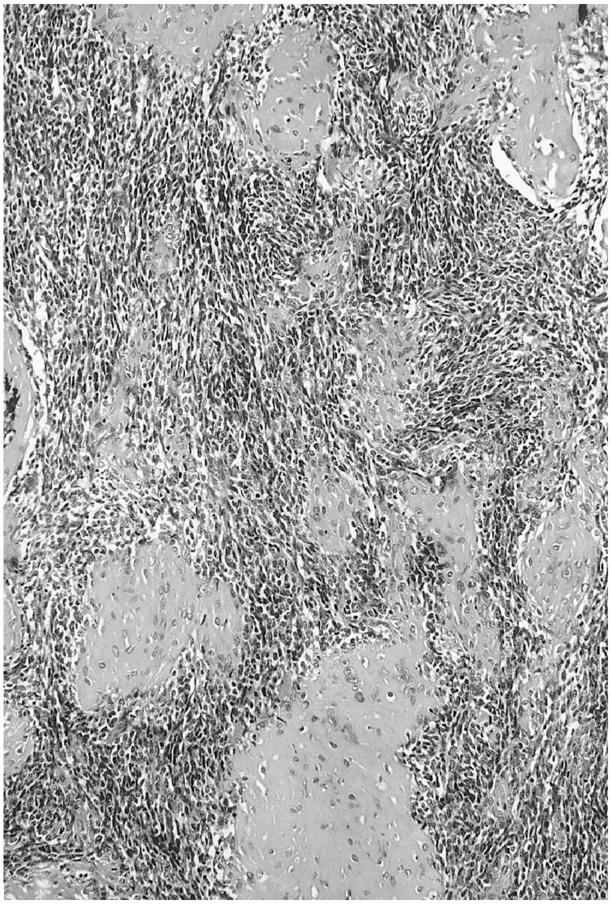

**Fig. 2.** *Extraskeletal osteosarcoma of orbit, with predominant spindle cell sarcomatous areas interspersed with neoplastic osteoid and focus of calcification (H&E; low power views).*

out excision of bone or muscle. Post-operative evaluation showed normal function of all ocular muscles.

On macroscopic examination the tumour was 3 × 3.5 cm in size, well circumscribed and firm in consistency. It showed greyish white areas on cut sections. Microscopy showed predominantly spindle cell sarcomatous areas interspersed with abundant neoplastic osteoid. Focal areas of neoplastic cartilage and osteoid with calcification were also evident (Figs 2–4).

Post-operative scanning showed no evidence of residual disease in the orbit, and CT scans of the chest and abdomen and radionucleide bone scan showed no evidence of disease elsewhere. The patient was unwilling to undergo orbital exenteration or local radiotherapy. He received six cycles of combination chemotherapy with cisplatin, ifosfamide and doxorubicin and is alive and free of disease 2 years after diagnosis.

### Discussion

Extraskeletal osteogenic sarcoma is unusual and reportedly accounts for only 2–4% of osteosarcomas.[1–4] Patients with extraskeletal osteosarcomas usually present in the fourth and fifth decades of life in contrast to their osseous counterparts.[1–4] Some series report a male predominance, whereas others show no sex predilection.[3,5]

The extremities and girdles, especially lower, are most commonly involved.[5] There are also reports of such tumours involving the face, breast, abdominal wall, soft tissues of the back and retroperitoneum.[2,3,5] Fine and Stout reported a case of osteogenic sarcoma arising at the site of a vaccination scar.[5] Kauffman and Stout reported the case of an 11-year-old boy developing orbital extraskeletal osteosarcoma, following radiation therapy for retinoblastoma in infancy.[6] The role of trauma in the development of extraskeletal osteosarcomas is controversial, though a history of trauma can be elicited in 13% of patients with these tumours.[3] The insidious evolution of osteogenic sarcoma in myositis ossisficans was described by Shanoff *et al.*[7] and around 16% of extraskeletal osteosarcomas have developed in myositis ossificans.[5]

Radiotherapy is known to predispose to the development of both soft tissue and bone sarcomas. In a series by Sordillo *et al.*,[2] 10% of the patients had previous irradiation to the area where extraskeletal osteogenic sarcomas developed, with a median interval of 15 years for development of these tumours. Our patient gave no history of trauma or previous radiotherapy to the eye.

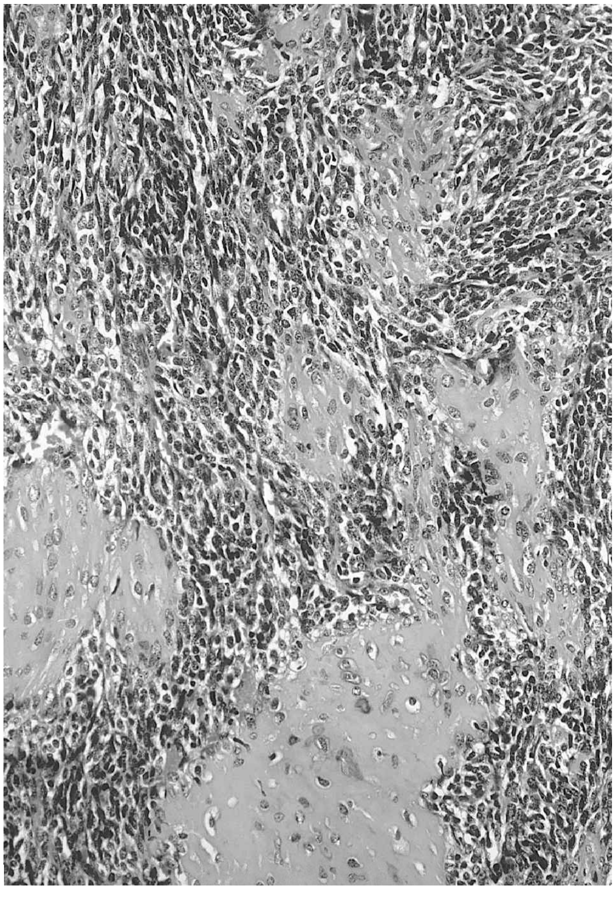

**Fig. 3.** *Extraskeletal osteosarcoma of the orbit, showing neoplastic osteoid (H&E; high power view).*

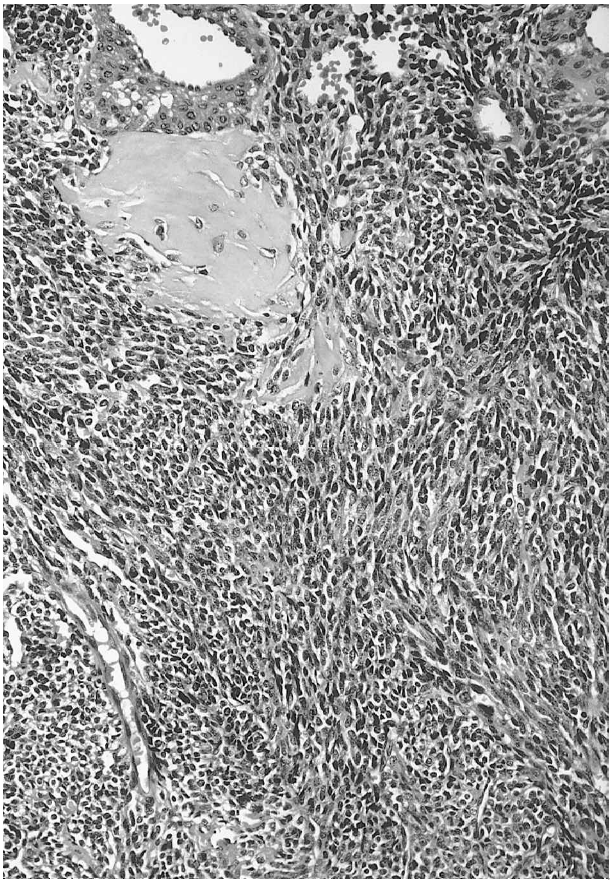

**Fig. 4.** *Extraskeletal osteosarcoma of the orbit, showing spindle cell area (H&E; high power view).*

Localised pain, swelling and oedema are the commonest presenting symptoms.[3] Duration of symptoms vary from weeks to years and most series report an average duration of 4–6 months.[2,3]

Radiologically the lesion presents as a soft tissue mass with spotty to massive calcification, without evidence of bony involvement.[2,4] Findings on magnetic resonance imaging are non-specific, though most tumours were heterogeneous and hyper-intense to muscle on T1-weighted imaging, and demonstrated high signal intensity on T2-weighted imaging.[8]

Microscopic features of extraskeletal osteogenic sarcomas are similar to those of the primary osseous variety, though most tumours are poorly differentiated and of high grade.[3] Variations in the amount of osteoid, cartilaginous and fibrous tissue have prompted most authors to classify the tumours as osteolytic, osteosclerotic or chondroblastic.[4,5] Fibroblastic zones generally show small uniform spindle cells. Large and pleomorphic cells and interlacing bundles of spindle cells are occasional findings.[3,9] Vascular invasion of the tumour is rare, and areas of necrosis are seen in some specimens.[3]

Primary extraskeletal osteosarcomas have a very aggressive natural history and local recurrences are common after incomplete excision, especially without additional radiotherapy.[2,9] Most patients die from metastatic disease, the common sites of involvement being lung, regional lymph nodes and bone.[5,10–12]

Treatment of these tumours has traditionally been radical surgery with or without additional radiation. Various chemotherapy protocols have been used in advanced and metastatic disease and the outcome was uniformly poor. In the series by Sordillo *et al.*,[2] four of the five patients who received adjuvant chemotherapy after surgical excision of recurrent or metastatic disease were long-term survivors, suggesting that chemotherapy may be of value in an adjuvant setting.

With the increasing use of chemotherapy, organ preservation could become feasible in patients with extraskeletal osteosarcoma. The use of pre-operative intra-arterial adriamycin infusion and gel embolisation followed by wide excision of the tumour was reported by Dhillon *et al.* for achieving limb preservation.[12] Our experience also suggests that preservation of organ/function could be achieved with limited surgery and chemotherapy. Chemotherapy schedules like CyADIC (cyclophosphamide, doxorubicin, and dacarbazine) or MAID (Mesna, ifosfamide, doxorubicin and dacarbazine) are currently being tried to evaluate response of these tumours prior to surgery.[13] Though definitive guidelines cannot be made, current data suggest that the optimal management of these aggressive tumours involves the use of chemotherapy and organ-preserving surgery with or without additional radiotherapy.

### Acknowledgement

The authors gratefully acknowledge Dr. Cyril Fisher, at the Department of Pathology, The Royal Marsden NHS Trust, London, for kindly reviewing the histopathology slides and confirming the diagnosis.

### References

1 Allan CJ, Soule EH. Osteogenic sarcoma of the somatic tissues—Clinicopathologic study of 26 cases and review of literature. *Cancer* 1971; 27:1121–33.

2 Sordillo P, Hajdu SI, Magill GB, *et al.* Extra skeletal osteosarcoma—A review of 48 cases. *Cancer* 1983; 51:727–34.

3 Rao U, Cheng A, Didolkar MS. Extraosseous osteogenic sarcoma—Clinicopathological study of eight cases and review of literature. *Cancer* 1978; 41:1488–96.

4 Pach GT, Braund RR. Development of sarcoma in myositis ossificans. *J Am Med Assoc* 1942; 119:776–80.

5 Fine G, Stout AP. Osteogenic sarcoma of the extraskeletal soft tissues. *Cancer* 1956; 9:1027–43.

6 Kauffman SL, Stout AP. Extraskeletal osteogenic sarcomas and chondrosarcomas in children. *Cancer* 1963; 16:432–9.

7 Shanoff LB, Spira M, Hardy B. Myositis ossificans: evolution to osteogenic sarcoma. Report of a histologically verified case. *Cancer* 1967; 113:537–41.

8 Varma DG, Ayala AG, Guo SQ, *et al.* MRI of extraskeletal osteosarcoma. *J Comput Assist Tomogr* 1993; 17:414–7.

9 Chung EB, Elzinger FM. Extraskeletal osteosarcoma. *Cancer* 1987; 60:1132–42.

10 Boyer CW, Navin JJ. Extraskeletal osteogenic sarcoma—A late complication of radiation therapy. *Cancer* 1965; 8:628–33.

11 Doiud TM, Moser RP Jr, Gindici MA, *et al.* Extraskeletal osteosarcoma of the thigh with several suspected skeletal metastases and extensive metastases to the chest. *Skeletal, Radiol* 1991; 20:628–32.

12 Dhillon KS, Suntharalingam S, Maurer HJ. Extraskeletal osteosarcoma of the thigh. *Med J Malaysia* 1993; 48:453–6.

13 Patel SR, Benjamin RS. Primary extraskeletal osteosarcoma—experience with chemotherapy. *J Natl Cancer Inst* 1995; 87: 1331–3.
